# Perceptual Learning in the Absence of Task or Stimulus Specificity

**DOI:** 10.1371/journal.pone.0001323

**Published:** 2007-12-19

**Authors:** Ben S. Webb, Neil W. Roach, Paul V. McGraw

**Affiliations:** Visual Neuroscience Group, School of Psychology, University of Nottingham, Nottingham, United Kingdom; Istituto di Neurofisiologia, Italy

## Abstract

Performance on most sensory tasks improves with practice. When making particularly challenging sensory judgments, perceptual improvements in performance are tightly coupled to the trained task and stimulus configuration. The form of this specificity is believed to provide a strong indication of which neurons are solving the task or encoding the learned stimulus. Here we systematically decouple task- and stimulus-mediated components of trained improvements in perceptual performance and show that neither provides an adequate description of the learning process. Twenty-four human subjects trained on a unique combination of task (three-element alignment or bisection) and stimulus configuration (vertical or horizontal orientation). Before and after training, we measured subjects' performance on all four task-configuration combinations. What we demonstrate for the first time is that learning does actually transfer across both task and configuration provided there is a common spatial axis to the judgment. The critical factor underlying the transfer of learning effects is not the task or stimulus arrangements themselves, but rather the recruitment of commons sets of neurons most informative for making each perceptual judgment.

## Introduction

Practice improves our ability to detect and distinguish sensory stimuli. Perception of most, if not all, sensory attributes improves with practice [1]–[9] and the perceptual benefits can be realized over many years [10]. The extent to which “perceptual learning” generalizes to novel, untrained stimulus attributes is very much dependent upon the task demands of training [11]. Learning of relatively coarse stimulus discriminations, for example, that requires little perceptual effort tend to generalize to untrained stimuli. Yet, when making challenging sensory judgments that demand focused attention, practice-induced improvements in perceptual performance are tightly coupled to the particular task and stimulus arrangement used during the initial training period [11].

The nature of specificity and generalization in perceptual learning is generally regarded to signify which population of neurons is solving the task and encoding the learned stimulus [4], [12]–[15]. For example, the specificity of perceptual learning for low-level visual characteristics, such as orientation and retinal position, has led to the view that tuning of individual cortical neurons to the ‘learned’ stimulus or weighting of synaptic connections between neurons in early visual cortex must be malleable or ‘plastic’ [15]–[23]. Precisely how and at what level(s) of visual cortex this experience-dependent plasticity is implemented remains controversial. According to one popular theoretical account of visual perceptual learning [12], the learning process begins at the top of the cortical visual hierarchy and works backwards searching for the most informative neurons to solve the prevailing sensory objective. Coarse, or ‘easy’, visual discriminations are learned by neurons with large receptive fields and broad stimulus tuning at advanced stages of cortical visual processing. Challenging, fine discriminations, on the other hand, are learned by neurons at earlier stages of cortical visual processing, where receptive fields are tightly tuned for retinal position and visual stimulation. It is the narrow tuning of these neurons which is thought to limit the transfer of learning effects to a restricted range of stimulus attributes [12].

**Figure 1:**
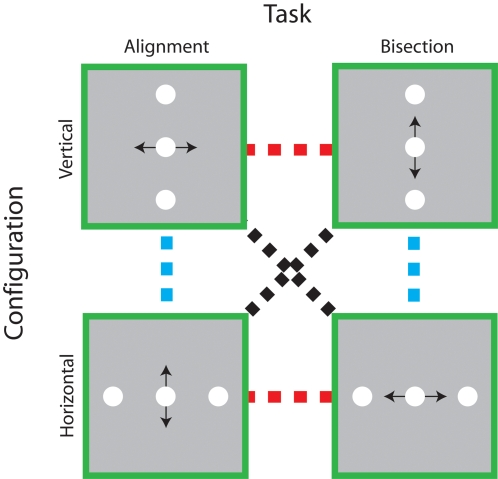
Decoupling the task and stimulus specificity of positional learning. Schematic illustrations of each of the task and stimulus configurations used in the experiment are shown in the green boxes. In each case, the black arrow indicates the spatial axis of the required positional judgement. To characterise the specificity of perceptual learning, subjects' performance was measured on all four task-configuration combinations before and after they received training on one of them. In principle, trained improvements in performance on particular task-configuration combinations could be tightly coupled to the task (dashed blue lines), the stimulus configuration (dashed red line) or the spatial axis of the judgement (dashed black lines).

Trained improvements on fine visual discriminations are often specific for stimulus orientation, direction of motion, retinal location, trained eye [24]–[31], and do not transfer between very similar tasks [32]–[34], even when the stimulus elements share a common retinal location [34]. Here we systematically decouple task- and stimulus-mediated components of trained improvements in perceptual performance and show that neither alone provides an adequate description of the learning process. The two-dimensional paradigm (shown in Fig 1A) enabled us to dissect task- and stimulus-coupled improvements in judgments of spatial position, a visual task that is particularly amenable to perceptual learning [4], [13], [18], [26], [31], [32], [34]–[37]. If perceptual learning is tightly coupled to task performance with little regard for the spatial pattern of retinal stimulation, improvements on a positional alignment or bisection task should transfer between different orientations (dashed blue line), but not between tasks (dashed red line). On the other hand, if training improves subjects' sensitivity to particular stimulus configurations regardless of the current task demands, improvements with vertical or horizontal oriented stimuli should transfer between different tasks (dashed red line). An intriguing alternative to these two potential outcomes is that learning selectively transfers along the spatial axis of the positional judgment itself, independently of either task or stimulus configuration (dashed black line).

## Results

Our first objective was to determine the extent to which practice enhanced visual position estimates within each training condition. Subjects were assigned to one of four groups, each of which trained for eight days on a unique combination of task (three-element alignment or bisection) and configuration (vertical or horizontal orientation), schematically represented in Figure 1. We obtained daily estimates of their performance (positional thresholds) at each of the separations between the central and reference elements. We expressed the data collected in an individual session as the proportion of trials on which the central element was judged to be rightward (Vertical alignment or Horizontal Bisection) or upward (Horizontal alignment or Vertical Bisection) of the reference elements. From logistic fits to these data, we estimated positional thresholds for each separation.

**Figure 2:**
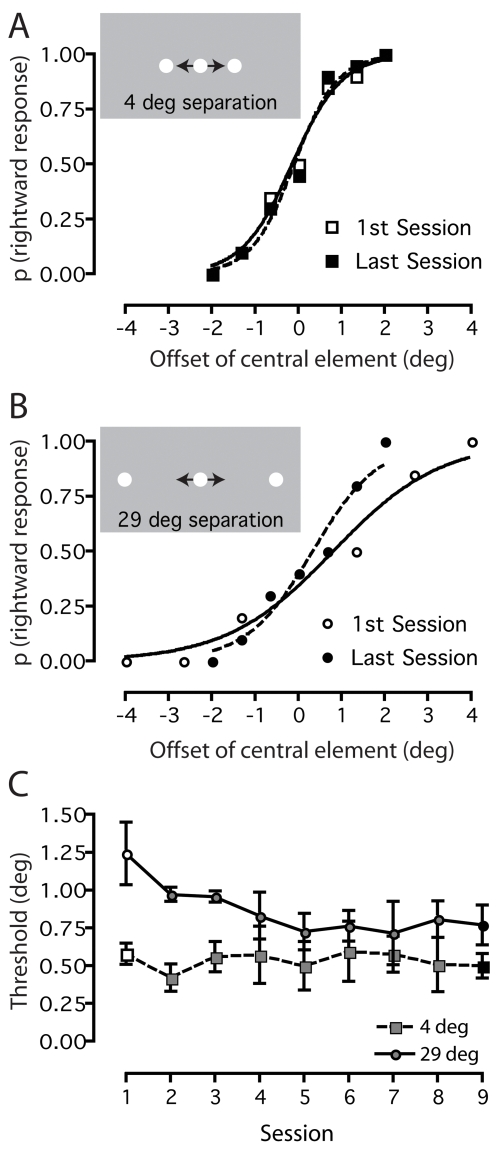
Quantifying positional learning. Example individual results are shown for a typical subject trained on the horizontal bisection task. a) A comparison of psychometric functions obtained on the first and last day reveals negligible learning at the smallest stimulus separation (4 deg). b) In contrast, a noticeable steepening of the psychometric function is evident at the largest stimulus separation (29 deg), indicating a learned improvement in task performance. c) Positional thresholds derived from the individual's psychometric functions, plotted as a function of session for the two extreme stimulus separations.

Figure 2 illustrates how we derived positional thresholds at two separations for an individual subject who trained on the horizontal bisection task. In Figure 2A, there is very little difference between the slopes of the psychometric functions (a measure of positional threshold) obtained during the first (open squares) and last (black squares) training sessions, indicating that training did not improve this subject's bisection thresholds at the smallest (4 deg) separation. The same analysis at the largest (29 deg) separation reveals a different picture (Fig 2B): the slope of the function measured during the last training session (black circles) is substantially steeper than that measured during the first session (open circles), showing that perceptual training reduced bisection thresholds at large separations. Figure 2C shows the bisection threshold estimates obtained at both separations during all training sessions. This plot shows clearly that perceptual training systematically reduces bisection thresholds at a large separation (circles), but has negligible effects on them at the smallest separation (squares).

**Figure 3:**
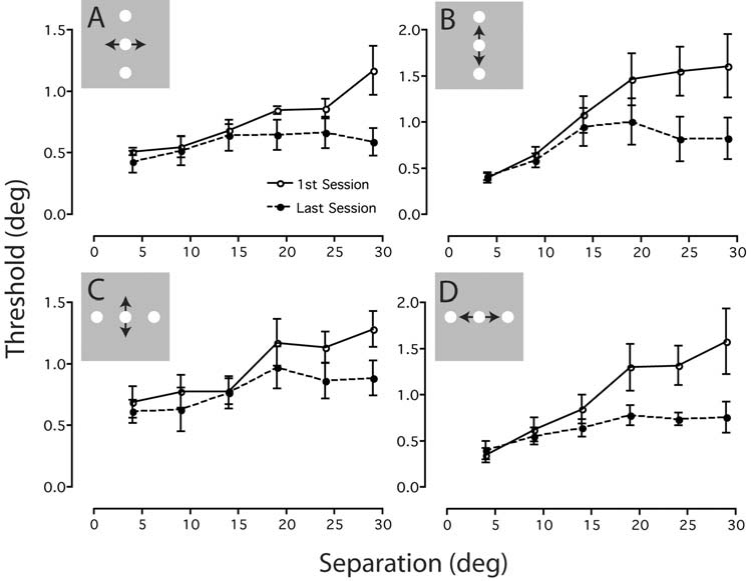
Positional learning for each task and stimulus configuration. Mean positional thresholds obtained on the first and last days for groups trained on a) vertical alignment, b) vertical bisection, c) horizontal alignment and d) horizontal bisection. Learned improvements in task performance are consistently seen for large, but not small, stimulus separations. Error bars are SEM.

To see if this pattern of learning held for each training condition, we calculated group averaged positional thresholds (±SEM) in the first and last session for each task-configuration combination. Figure 3 shows the results of this analysis plotted as a function of separation. For the alignment task, thresholds obtained during the first session at small separations (≤14 deg) are almost independent of separation, but for larger separations increase in proportion to separation (open circles; Fig 3A–B). For the bisection task, thresholds obtained during the first session increase roughly in proportion to separation at all values of separation (open circles; Fig 3C–D). Yet the effects of training on both tasks are manifested in the last session as a marked reduction in thresholds only at large (>14 deg) separations between the central and reference elements (black circles; Fig 3A–D). This result sits comfortably with the notion that positional sensitivity at small and large separations are governed by different neural processes [38], [39].

**Figure 4:**
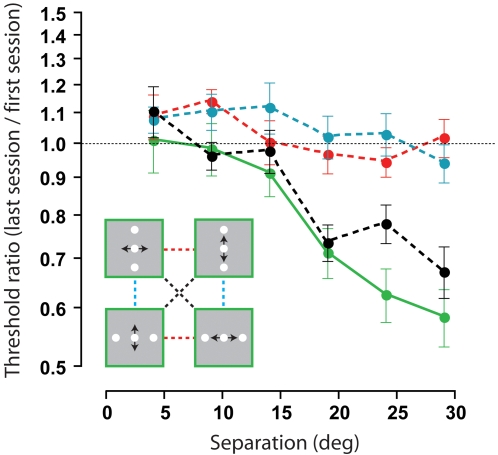
Transfer of learned improvements between task and stimulus configurations. Ratio of mean positional thresholds obtained on the first and last days, collapsed across conditions and plotted as a function of stimulus separation. Trained improvements in performance did not transfer to different configurations (dashed blue line) or tasks (dashed red line), but did transfer between task-configuration combinations when there was common spatial axis to the judgement (dashed black line). For comparison, the solid green line shows averaged learned improvements obtained within each task-configuration combination. Error bars are SEM.

Figure 4 shows the magnitude of perceptual learning expressed as the ratio of subjects' performance on the last and first day as a function of separation between the central and reference elements. The solid green line confirms how training within task (collapsed across all task-configuration combinations) only reduced positional thresholds when there were large (>14 degree) separations between the central and reference elements. In agreement with previous work [13], [26], [34], perceptual training did not improve positional sensitivity independently of the stimulus orientation (dashed blue line) or task demands (dashed red line). Both results are consistent with the view that trained improvements in challenging sensory discriminations do not transfer between different tasks or stimulus configurations [11], [12]. What we demonstrate for the first time is that learning does actually transfer across both task and configuration provided there is a common spatial axis to the judgment (dashed black line). This pattern of transfer suggests that for relative position estimates, neurons which encode the spatial axis of the judgment rather than the stimulus orientation or task *per se* are most informative for the learning process.

## Discussion

The hallmark of visual perceptual learning is its specificity for simple visual attributes, frequently being tightly coupled to the trained retinal position [28], [40]–[43], orientation [24], [28], [42], spatial frequency [24] and size of a visual pattern [24], [40], [44]. It is widely believed that stimulus-coupled learning reflects some form of experience-dependent plasticity at the cortical level which encodes the stimulus itself [15]–[23], [42]. For example, experience-dependent plasticity can arise as early primary visual cortex [16], [19], [21]–[23], where neurons encode simple stimulus attributes such as orientation [45], [46] and spatial frequency [47], [48].

In agreement with previous work [13], [26], [34], we have shown that perceptual training does not improve positional sensitivity independently of the stimulus configuration and task. However, our findings demonstrate that characterizing the specificity of learning in terms of the arrangement of stimuli and/or the type of perceptual judgment is potentially misleading. Specifically, we demonstrate that learned improvements in positional sensitivity transfer to conditions in which both these factors have been altered, provided that the spatial axis of the judgment remains constant. These findings most likely reflect the recruitment of common sets of neurons that are informative for making each judgment. Indeed, recent physiological evidence suggests that learned-induced changes in the neural population response are specific to neurons that provide the most information for solving the learned task [49]. Even though stimulus configuration and the type of task performed undoubtedly play a role in dictating which neurons are most useful for making a given perceptual decision, neither factor alone is a perfect predictor of the specificity of perceptual learning.

Visual representations that mediate fine discriminations of stimulus position reside at early levels of cortical visual processing, where neuronal receptive fields are small and retinotopically organized. Feasibly, learned improvements in making positional judgments along a spatial axis might reflect plastic changes at this early stage of visual processing or, alternatively, at later cortical stages where positional information is combined. A strong indication of how these neural plastic changes might arise is provided by our observation that the magnitude of learning increases with the separation between the central and reference elements. It is well established that the accuracy of positional estimates is inversely proportional to stimulus separation [38], [50], [51]. There are at least two putative neural mechanisms that could explain this separation-dependent degradation of positional acuity. The first is that positional acuity is mediated by independent mechanisms at small and large separations [52]–[54]. For example, in an alignment task, observers could utilize the output of neurons that encode neighboring stimulus elements when they are in close proximity [55], [56] and the retinal position or ‘local sign’ of each element when they are widely separated [57], [58]. Although this scheme could explain why we observed separation-dependent learning, it does not explain the specific transfer of learning across tasks involving judgments along a common spatial axis. In addition, it is difficult to reconcile with previous studies that have reported substantial positional learning effects at small stimulus separations [e.g. 13], [18], [20], [26], [31], [32], [34], [36], [37].

The second putative mechanism is that there is a gradual shift to lower spatial scales of analysis with increasing separation [38], [50]. That is, increasingly larger receptive fields have to be recruited to encode the stimulus elements as separation (and eccentricity) increases. Within this framework, positional learning would cause a shift to increasingly smaller spatial scales of analysis at large separations. The reason why the magnitude of learning increases with stimulus separation is there is much more scope to move to finer spatial scale of analysis at larger separations, whereas at small separations the receptive fields are already operating at the optimal spatial scale of analysis [38]. To explain the observed patterns of transfer, this scheme requires that learning-induced changes in spatial scale are tightly coupled to the spatial axis of the judgment but not the retinal position of the stimulus elements. Work is underway in our laboratory to test this possibility.

This putative mechanism also enables us to reconcile our results with previous work demonstrating large learning effects at small and medium stimulus separations. To the best of our knowledge, all of the previous studies that have demonstrated substantial positional learning effects at small separations have either used broadband stimuli (e.g. dots, lines, bars) or medium to high frequency sinusoidal gratings [13], [18], [20], [26], [31], [32], [34], [36], [37], [59]–[62]. Because of the frequency content of these stimuli, there is much more scope to recruit receptive fields with finer spatial scales to improve positional thresholds. With the Gaussian luminance patches that we have used here, performance at small and medium stimulus separations is largely independent of separation and limited by the low spatial frequency content of the stimulus envelope [38]. This mechanism prevents the recruitment of fine spatial scale receptive fields and ultimately places an upper limit on the magnitude of positional learning at small separations. It therefore explains the small learning effects that we observed at small and medium separations.

In principle, the transfer of positional learning could be explained by training induced enhancements in “absolute” positional localization of the central element. In the experimental arrangement, we did not set out to limit this possibility because previous research has highlighted the marked retinal specificity of visual learning [28], [40]–[43]. Therefore, if we had jittered the position of our stimulus configurations on a trial-by-trial basis and not observed any learning, it would have been impossible to distinguish whether this was due to a genuine lack of perceptual improvement or simply a consequence of jittering the retinal position of the stimulus. It seems unlikely that absolute position cues substantially contributed to the transfer of learning because absolute positional thresholds at a given eccentricity can be up to four times worse than the best relative position thresholds [58], [63]. Moreover, both our learning and transfer effects are separation-dependent, arguing against the notion that subjects used the absolute position of the central element or the edges of the monitor to make their judgments.

Our results necessitate a new interpretation of the concept of stimulus specificity so often shown in perceptual learning studies. The critical factor in the transfer of learning effects between tasks is not the task or stimulus arrangement, but rather the recruitment of common sets of neurons most informative for making each perceptual judgment. Although we have used a two-dimensional position design to make this distinction, it is unlikely that this principle is peculiar to the visuo-spatial domain.

## Materials and Methods

### Subjects

24 subjects (21–33 years; 13 males, 11 females) with normal or corrected-to-normal visual acuity participated. All were naïve to the specific purposes of the experiment. We obtained written consent from all subjects who were free to withdraw from the study at any time.

### Stimuli

Stimuli were generated on an *Apple Macintosh G5* using custom software written in Python [64] and displayed on a gamma corrected *LaCie Electron 22 Blue IV* monitor with a resolution of 1920×1440 pixels, viewing distance of 23.8 cm and update rate of 100 Hz. The stimulus elements consisted of three circular patches with Gaussian luminance profiles (standard deviation 0.33 degrees) on a uniform background (luminance ∼50 cd/m^2^), one positioned centrally at fixation and flanked by two others at a range of separations. The separation between the reference elements and central element ranged between 4–29 degrees in 5 degree steps. On each trial, stimuli were presented for 0.2 sec and separated by a 0.5 sec interval containing a blank screen of mean luminance.

### Procedure

The experimental protocol was approved by the local ethics committee at the School of Psychology, University of Nottingham. Subjects viewed the display with their head held in a fixed position with a chin and forehead rest. Prior to the start of the experiment, we obtained an estimate of subjects' alignment and bisection thresholds at each separation using an adaptive staircase procedure, with a total of 50 presentations. This estimate determined the step size and range of values in the Method of Constant Stimuli, used throughout the remainder of the experiment to estimate positional thresholds. For each condition, positional thresholds at six separations were estimated in a single run of 420 randomly interleaved trials (20 trials per point on the psychometric function). Subjects did not receive any feedback on their performance.

On the first day of the experiment, we measured subjects' thresholds on all four combinations of task (three-element alignment and bisection) and configuration (vertical and horizontal orientation) in a random order. In the alignment task, subjects had to indicate whether the central element was offset to the left or right from vertically separated reference elements (vertical alignment) or above and below horizontally separated reference elements (horizontal alignment). In the bisection task, they had to judge whether the central element was closer to the upper or lower reference element (vertical bisection) or closer to the right or left reference element (horizontal bisection).

Subjects were pseudo-randomly assigned to one of four groups, each of which trained for eight days on one combination of task and configuration. During the training period, we obtained daily estimates of their positional thresholds at each separation using the methods described above. On the last day of the experiment, we re-measured subjects' thresholds on all four combinations of task and configuration in a random order.

### Data analysis

We expressed the data as the proportion of trials on which subjects judged the central element to be rightward (Vertical alignment or Horizontal Bisection) or upward (Horizontal alignment or Vertical Bisection). To estimate positional thresholds, we fitted a logistic equation to these data of the form:
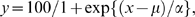
(1)where *y* is the proportion of rightward or upward positional judgements, *μ* is point of subjective equality (the 50% point on the psychometric function), and *α* is an estimate of alignment or bisection threshold.
